# Optimizing Radiation Patterns of Thinned Arrays with Deep Nulls Fixed through Their Representation in the Schelkunoff Unit Circle and a Simulated Annealing Algorithm

**DOI:** 10.3390/s22030893

**Published:** 2022-01-24

**Authors:** Mateo Raíndo-Vázquez, Aarón Ángel Salas-Sánchez, Juan Antonio Rodríguez-González, María Elena López-Martín, Francisco José Ares-Pena

**Affiliations:** 1 Radiating Systems Group, Department of Applied Physics, Faculty of Physics, University of Santiago de Compostela, E-15782 Santiago de Compostela, Spain; mateo.raindo@rai.usc.es (M.R.-V.); aaronangel.salas@usc.es (A.Á.S.-S.); ja.rodriguez@usc.es (J.A.R.-G.); 2 Department of Morphological Sciences, Faculty of Medicine, University of Santiago de Compostela, E-15782 Santiago de Compostela, Spain; melena.lopez.martin@usc.es

**Keywords:** linear array antennas, array thinning, Schelkunoff unit circle, null fixing, directivity

## Abstract

The present work develops an innovative methodology for fixing deep nulls in radiation patterns of symmetrical thinned arrays while maintaining a low side lobe level (SLL) and a high directivity, implementing an optimization strategy based on the simulated annealing algorithm (SA). This procedure optimizes a cost function that has a term for each characteristic of the desired radiation pattern and can distinguish between the deep nulls and the filled ones depending on whether they are on the Schelkunoff unit circle or not. Then, a direct extension of the methodology for planar arrays based on the separable distribution procedure is addressed. Consequently, some examples with half-wavelength spacing are presented, where the fixing of one, two, or three deep nulls in arrays of 40, 60, and 80 elements are illustrated as well as an extension to a 40 × 40-element planar array with rectangular grid and rectangular boundary, with two deep nulls fixed on each one of its main axes. Additionally, a comparison of the obtained results with a genetic algorithm (GA) alternative is performed. The main advantage of the proposed method is its ability to fix deep nulls in the radiation patterns, while maintaining an easy feeding network implementation.

## 1. Introduction

The performance of antenna arrays regarding low side lobe level (SLL) and high directivity, at the same time that there is a far field depression on a certain range of their radiation pattern, represents a very interesting concern on antenna array designs with impact not only on radar [1] and space applications [2], but also in the new design strategies for the future 5G communication deployment [3,4], where options such as metamaterial-based antenna designs are becoming of high interest in recent literature [5]. In this same framework, but concerning channel coding design, low density parity check (LDPC) coding is attracting great attention for improving transmission reliability [6,7].

Nowadays, suppressing power in precise angular regions of the radiation pattern of high-performance antennas still represents a challenge for the antenna community. In such a way, to have control over these above-mentioned parameters by altering the element excitations and configuration, results in a complex problem with a variety of approaches [8,9,10]. Numerical optimization methods are commonly used in order to find the appropriate solution with the desired characteristics without extensively checking all the possibilities in the solution space. 

Array thinning is a technique for the design of antenna arrays, based on removing (or turning off) some elements of an array without significantly changing its beamwidth [8] (p. 92). Consequently, as is well-known, the directivity of the array will be directly related to the area of illumination of the aperture, therefore, a reduction in a fraction of the level at the filled case is expected, by means of the elements removed. In such a case, it may be possible to exploit this strategy to build moderate high directive arrays. In this framework, after analyzing the different possibilities regarding the cost savings of complex power divider networks, uniform illumination represents a potentially good alternative for feeding these solutions. Therefore, by restricting the excitation possibilities from a continuous problem to a binary one, the number of solutions is decreased to a finite combination of zeros and ones. 

Therefore, when analyzing the literature, it can be seen that extensive studies lowering the SLL of the pattern while maintaining a high directivity in thinned arrays have been developed. For instance, examples of using genetic algorithms (GA) [11,12], fast Fourier transform techniques (FFT) [13], ant colony optimization (ACO) [14], differential algorithm (DA) [15], pattern search algorithms (PS) [16], and biogeography-based optimization (BS) [17] correspond to interesting approaches included within the literature.

At the same time, as has already been mentioned, avoiding interference with a receiving signal by suppressing the radiation pattern in a certain direction or range is also of great interest. Several approaches to this optimization problem have also been addressed in the literature from achieving a certain region of the radiation pattern under a desired level by altering the thinned pattern of the array [18] to using optimization algorithms in order to minimize the value of the radiation pattern in a certain direction, where examples based on GA [19] or the whale optimization algorithm (WO) [20] by altering the excitation amplitudes and phases or the interelement spacing can be reported.

On the other hand, the placement of deep, analytic, nulls of the radiation pattern is a very interesting approach since it represents a more accurate method of guaranteeing the presence of a null position on the radiation pattern. Examples of works achieving this placement via the control of the interelement spacing using particle swarm optimization (PSO) [21] can be highlighted. Alternatively, methods of controlling nulls through formulations involving changes in amplitude and phase of each element in the array [22,23] can be reported.

In the present work, thinned uniform arrays are proposed to maintain low cost and ease of implementation of the feeding network. To this aim, the use of the seminal work of Schelkunoff [24], in order to represent the roots of the array as in [25], is proposed to discern between the deep analytical nulls and the filled ones. Therefore, we can optimize our pattern by minimizing the distance from a desired deep null to the closest one from our pattern lying on the unit circle, while maintaining a high directivity and low SLL with the simulated annealing algorithm (SA). To the best knowledge of the authors, none of the previous methodologies described in the literature have simultaneously dealt with deep null fixing and array thinning in array pattern synthesis.

## 2. Materials and Methods

### 2.1. Linear Arrays

Let us consider an equally spaced linear array of *N* isotropic elements laid out along the z-axis. In such a case, the expression of the array pattern or array factor F(θ) follows [26]

(1) $$F\left(\theta \right)=\overset{N}{\underset{n=1}{\sum }}I_{n}e^{j\left(n-1\right)kd\;cos\left(\theta \right)}$$

where k is the wavenumber; d the spacing between the elements; $I_{n}$ the relative excitation of the n-th element; and θ is the polar angle.

### 2.2. Array Pattern Nulls

**Figure 1:**
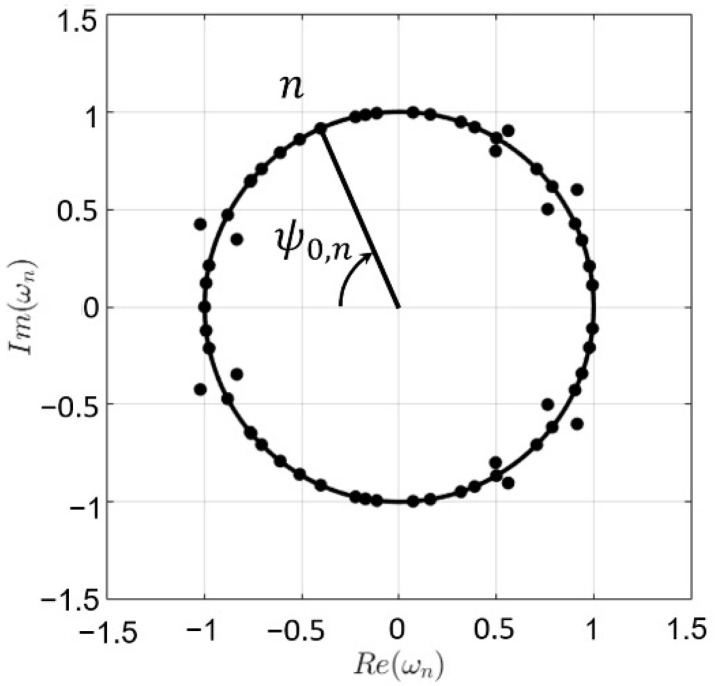
Example of the representation of a set of complex roots on the Schelkunoff unit circle.

The null of the far field radiation pattern of an equispaced linear array can be analyzed by means of a change of variables in (1) by introducing ψ=kd cos(θ) and $\omega =e^{j\psi }$ as is shown in [27]. Thus, the array factor becomes $F\left(\omega \right)=I_{N}f\left(\omega \right)$, where f(ω) is given by

(2) $$f\left(\omega \right)=\overset{N}{\underset{n=1}{\sum }}\frac{I_{n}}{I_{N}}\omega^{n}=\overset{N-1}{\underset{n=1}{\prod }}\left(\omega -\omega_{n}\right)$$

where $\omega_{n}$ is the n-th complex root of the polynomial produced by the array factor. In order to exploit this property and understand how null control can be developed, these roots can be represented on the complex ω plane [24] (see Figure 1).

All the roots that lie on the unit circle correspond to deep nulls of the pattern and, following the mathematical description for obtaining (2), the angular position of these complex roots is linked to the angular position on the far-field pattern by means of the expression $\psi_{0,n}=kd\;cos\theta_{0,n}$ as described in Figure 1. Therefore, the null fixing here proposed is implemented by calculating the roots of the relative excitation polynomial of the antenna and transforming the angular position ($\psi_{0,n}$) of the roots that lie on the unit circle to the angular position ($\theta_{0,n}$) on the radiation pattern. Then, for each selected null position, a term expressing the minimum distance from one of the actual pattern nulls to the desired null can be introduced within the cost function of an optimization strategy. 

### 2.3. Directivity of Linear Arrays

To evaluate the quality of the radiation pattern produced by a linear array, the peak directivity is calculated from the relative excitation vector itself, following the approach in [26] (pp. 153–154). More precisely, in the case of an isotropic equally spaced (d=λ/2) element pattern, the expression is simplified to

(3) $$D_{max}=\frac{{\left(\sum_{n=1}^{N}I_{n}\right)}^{2}}{\sum_{n=1}^{N}I_{n}^{2}}$$

where $D_{max}$ is the peak directivity. This simplification allows us to obtain the peak directivity of the linear arrays without calculating any integrals of the radiation pattern, drastically reducing the computation time. Therefore, particularly in the present work, uniform excitations were addressed aiming to a practical feasibility of the feeding network. In such a case, the directivity can be calculated simply by counting the number of active elements in the linear array. Therefore, in this work, the normalized peak directivity of the antenna array was calculated by comparing it to the directivity of the uniform solution (that is, with all the elements of the array turned on). Thus, this normalized peak directivity corresponds to the percentage of active elements, which is calculated dividing the directivity between the total number of elements of the linear array.

### 2.4. Optimization Strategy

In order to achieve different improvements in the radiation pattern such as lowering SLL, thinned antenna arrays are proposed here. Such kinds of arrays have some elements turned off in order to achieve some pattern properties. Therefore, each element can then be in two states: 0 (or turned off) and 1 (or turned on). The number of solutions or turned on-turned off combinations increases very fast with the number of elements as there are $2^{N}$ combinations for an N element array. We decreased this number from the beginning by restricting our study to symmetrical arrays to have $2^{N/2}$ possible solutions for an array of N elements. One last restriction imposed over the relative excitation vectors is that the last element of the arrays must be necessarily turned on to guarantee arrays of determined effective length and a certain main lobe width. In other words, the last element of the edge is always on, attending to the premise that a linear array of N elements with the last element turned off is actually a linear array of N−1 elements. This results in $2^{N/2-1}$ combinations for an array of N elements.

**Figure 2:**
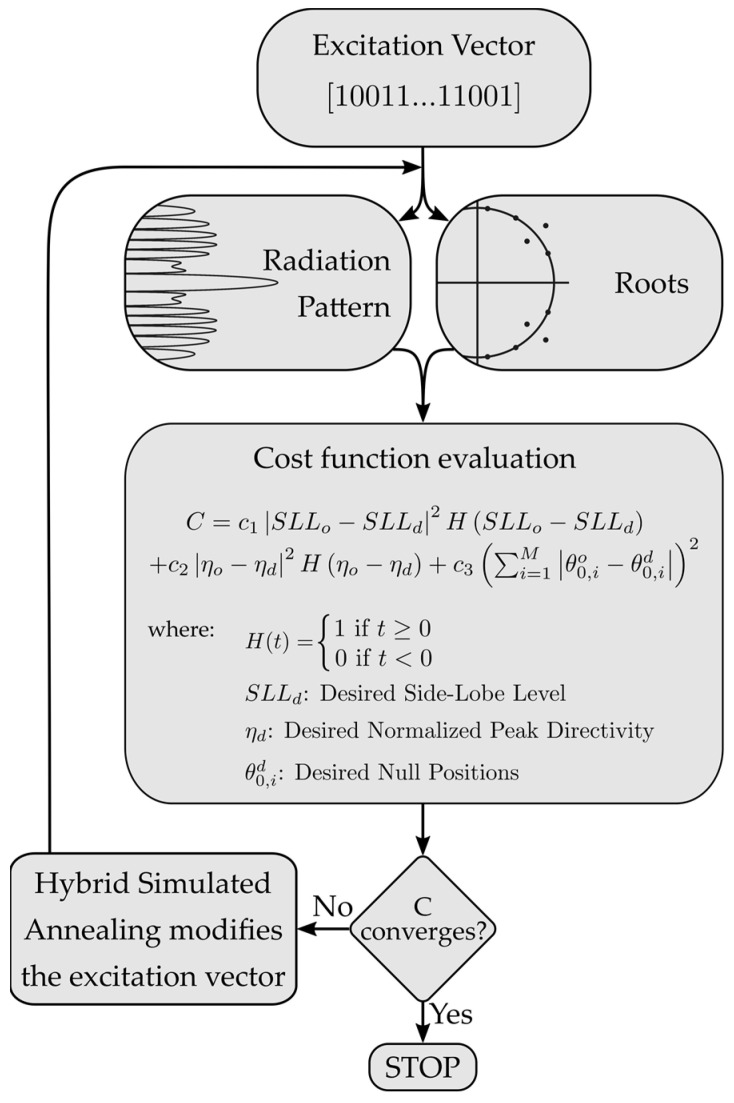
Flowchart of the optimization process based on a hybrid simulated annealing algorithm (SA). *M* represents the number of deep null angular positions to be fixed by the method.

Even with this simplification, the number of possible combinations is too large for big arrays, so checking all the possible combinations is discarded as it would not be an effective method. We need a way to navigate the solution space and look for the best combination without checking every solution. In such a way, a procedure has been envisaged, where a cost function (defined in Section 2.4.4) is minimized by means of a optimization algorithm iteratively until the radiation pattern has the desired characteristics, as shown in Figure 2.

Attending the optimization strategy, an algorithm that does not stop at a local minimum of the solution space and looks for the global best solution of the problem is needed. The chosen algorithm was hybrid SA [28], which is based both on the behavior of the downhill simplex, a local optimization method, and the SA, a global one. In order to explain the algorithm, both methods will be described first.

#### 2.4.1. Simulated Annealing

Simulated annealing is a global optimization method based on the behavior of a slowly settling thermodynamic system. A solid body that cools down slowly has a big solution space for particle configurations, on which the crystal lattice configuration is the one with the lowest energy (global solution of the problem), but other amorphous configurations (local minima of the solution space) are also possible. The system will only reach the state with the lowest energy if the temperature decreases slowly enough for each individual particle to find the perfect placement. 

The thermodynamic process is governed by the distribution of probability of Maxwell–Boltzmann, which relates to the probability that the system reaches a certain state with the internal energy of that state and the temperature of the system. In order to apply this method to other optimization problems, we must define a cost function that evaluates the “goodness’’ or energy of each state. We must also define the probability of jumping from a certain state to a newly generated one, based on the cost (internal energy) of each state and the temperature of the system, which will be reduced slowly as the program runs. The probability of going from a state with energy $E_{1}$ to one with energy $E_{2}$ can be described by

(4) $$p=e^{-\frac{\left(E_{2}-E_{1}\right)}{k_{B}T}}$$

where $k_{B}$ is the Boltzmann constant and T is the temperature of the system. As we can derive from the formula, if $E_{2}<E_{1}$, then the probability is larger than one, so the transition will always happen. If $E_{2}>E_{1}$, then the probability of jumping from state 1 to state 2 belongs to the interval (0,1) and decreases as the temperature decreases. This number will be compared to a randomly generated number between 0 and 1 and the transition between states will only take place if the calculated probability is bigger than the randomly generated number. 

#### 2.4.2. Downhill Simplex

**Figure 3:**
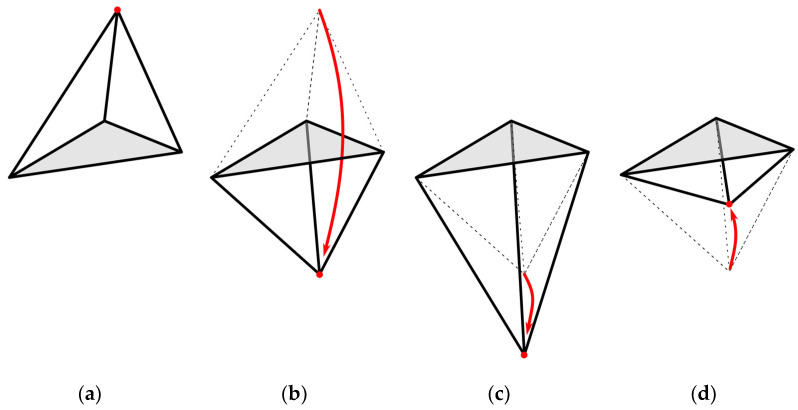
Simplex possible moving steps: (**a**) Simplex with the vertex with the highest cost represented with a red dot; (**b**) Reflection of the position of the vertex with the highest cost through the opposite face of the simplex; (**c**) Expansion of the simplex by moving the vertex away from the volume in the case that the reflected position achieved in (**b**) has a lower cost than the original position from (**a**); (**d**) Contraction of the simplex by moving the vertex toward the volume, in the case that the reflected position achieved in (**b**) has a higher cost than the original position from (**a**).

Downhill simplex is an iterative local optimization method based on the construction of successive geometrical figures denominated simplex. A simplex in a N-dimensional space is a polytope of N+1 vertices and the corresponding edges and faces linking them. In order to use this mathematical object for our optimization purposes, let us suppose a cost function f(x) we must optimize, where x is a vector of dimension N. A simplex is created with N+1 vertices of positions $x_{1},\;x_{2},\ldots \;x_{N+1}.$ The cost value for each vertex is now calculated and the one with the higher cost is moved to a new position through the opposite face of the simplex (see reflection in Figure 3b). If the cost of the new point is lower than the previous one, the algorithm will move it away further through the line, expanding the volume of the simplex (see expansion on Figure 3c). If the cost of the new position is higher, the algorithm will move it closer toward the simplex, reducing its volume (see contraction on Figure 3d). To summarize, a set of the different possible movements of the simplex is illustrated in Figure 3. This happens for every new configuration of the simplex, each time moving the vertex with the higher cost value toward a new point, and increasing or decreasing the total volume depending on how close to the solution each step takes us. When the volume of the simplex decreases to a minimum, it means it has found a local minimum of the function, because any step in any direction would only increase its volume.

#### 2.4.3. Hybrid Simulated Annealing

Consequently, as inferred from the previous subsections, the hybrid method used in this study mixes the local optimization behavior of the downhill simplex, introducing the temperature parameter from the SA, T, which also decreases slowly in this case, and allows the algorithm to find global minima and not get stuck in local ones. In this method, instead of comparing the cost of a vertex directly with the cost of the previous position, we included a term depending on the temperature. In such a way, the simplex will navigate the solution space more freely at the beginning and slowly settle into a minimum.

The algorithm includes a final strategy in order to avoid falling into a local minimum of the function, which is based on reiterations. We forced the algorithm to run (re-iteratively) five times, starting at a different randomized position each, so the final solution will be the best one achieved in all five re-iterations. This is an extra step that helps to guarantee the global minimum of the function.

In order to transform the continuous values resulting from the algorithm operations into binary values for the relative excitation vectors, a discretization method must be implemented. In our case, we summed the vector of small perturbations generated by the algorithm (positive and negative values) divided by a factor of 1000, to a uniform excitation vector with all values equal to 0.5. After this operation takes place, we looped over every element of the resulting vector and if its value was higher than 1, we set it to 1, and if it was lower than 0, we set it to 0. We then ran one last time over all the elements, setting its value to 1 if it was equal or higher than 0.5, and setting it to 0 if it was lower than 0.5.

#### 2.4.4. Cost Function Definitions

The cost function for each state must include all the parameters we want to optimize in our antenna arrays. As highlighted in Figure 2, we focused this study on three main factors: lowering the SLL, maintaining a high directivity, and fixing M nulls of the pattern at certain angles $\left(\theta_{0,1}^{d},\ldots ,\theta_{0,M}^{d}\right)$.

The cost term associated to the SLL is multiplied by a Heaviside function, defined in (5), where argument t is the difference between the obtained SLL (SLL_o_) and the desired one (SLL_d_) in order to only try to improve this aspect of the radiation pattern if the result was above a defined threshold.

(5) $$H\left(t\right)=\{\begin{array}{c} 1\;\;\;\mathrm{if}\;t\geq 0\\ 0\;\;\;\mathrm{if}\;t<0 \end{array}$$

Alternatively, concerning the directivity control of the far field pattern generated by the linear array, a Heaviside function concerned on looking solely for improvements in the case of being at a higher level of a desired directivity is proposed. This use becomes optional if certain directivity has to be fixed (for comparative purposes).

The complete mathematical expression of the cost function multiplies each contribution by a coefficient in order to change the relative importance of each term. In our case, the coefficients were chosen following the criterion of fixing a certain directivity with the most importance, then fixing null angular positions and finally lowering the SLL, as there was no point in our experiment to obtain a low SLL if we did not meet the other required specifications. Therefore, the final expression of the cost function is

(6) $$C=c_{1}{\left|{\mathrm{SLL}}_{o}-{\mathrm{SLL}}_{d}\right|}^{2}H\left({\mathrm{SLL}}_{o}-{\mathrm{SLL}}_{d}\right)+c_{2}{\left|\eta_{o}-\eta_{d}\right|}^{2}H\left(\eta_{o}-\eta_{d}\right)+c_{3}{\left(\overset{M}{\underset{i=1}{\sum }}\left|\theta_{0,i}^{o}-\theta_{0,i}^{d}\right|\right)}^{2}$$

where SLL_o_ and SLL_d_ are the obtained and desired SLL, respectively; $\eta_{o}$ and $\eta_{d}$ are obtained and desired normalized directivity on broadside; H(·) is the Heaviside function; *M* corresponds to the number of desired nulls to be fixed; $\theta_{0,i}^{o}$ and $\theta_{0,i}^{d}$ are the obtained and desired null position by means of their polar angles and, finally, $c_{1},\;c_{2},$ and$,\;c_{3}$ are the different weights of the cost function. As can be noted from (6), the cost function squares the terms in order to increase the convergence speed by enlarging the differences between costs of different input vector ($I_{n}$).

### 2.5. Extension to Planar Arrays: Separable Distributions

Once we obtained optimized linear arrays that maximize the SLL and directivity with a certain number of fixed nulls in the pattern, we can synthesize a rectangular grid and rectangular boundary planar array with separable distributions by placing one optimized array on the x-axis and another on the y-axis, achieving the value for the relative excitation of the rest of the elements of the planar array by multiplying the values of the elements in the x and y projections. By using this method, the relative excitation amplitude of each element of the planar array is given by:

(7) $$I_{mn}=I_{m}\times I_{n}\;$$

where m and n are the indices in the x and y directions for the elements in the planar array and $I_{m}$ and $I_{n}$ are the relative excitation vectors for the linear arrays placed in the x-axis and y-axis, respectively.

This method allows us to fix nulls in the $\varphi =0^{\circ }$ and $\varphi =90^{\circ }$ planes (as the radiation pattern in these planes will correspond to the ones from each introduced array) without losing the binary property of each element relative excitation. The main advantage of the separable distributions is the feeding network practical implementation, which can be simplified to a set of equal linear arrays fed from the left side of the planar array as described in [29]. 

The array factor considering a separable planar array synthesized from two linear symmetrically excited arrays lying on the x−y plane is given by the expression from [26] (p. 207):

(8) $$F\left(\theta ,\;\varphi \right)\;=4\overset{N_{x}}{\underset{m=1}{\sum }}\overset{N_{y}}{\underset{n=1}{\sum }}I_{m}I_{n}cos\left[\frac{\left(2m-1\right)}{2}kd_{x}sin\theta cos\varphi \right]cos\left[\frac{\left(2n-1\right)}{2}kd_{y}sin\theta sin\varphi \right]\;$$

where $N_{x}$ and $N_{y}$ are half of the elements of the arrays on the x and y-axis, respectively; $I_{n}$ and $I_{m}$ are the relative excitation patterns for the arrays; $d_{x},\;d_{y}$ are the interelement spacings of x and y directions; and θ and φ are the polar and the azimuth angles, respectively.

In order to calculate the peak directivity of the planar arrays, we used the following expression extracted from [26] (p. 205), as the simplified Equation (3) is only valid for linear arrays:

(9) $$D\left(\theta_{0},\varphi_{0}\right)=\frac{4\pi F\left(\theta_{0},\varphi_{0}\right)F^{*}\left(\theta_{0},\varphi_{0}\right)}{\int_{0}^{2\pi }\int_{0}^{\pi /2}F\left(\theta ,\varphi \right)F^{*}\left(\theta ,\varphi \right)sin\left(\theta \right)d\theta d\varphi }$$

where $\left(\theta_{0},\varphi_{0}\right)$ is the angular position of the maximum radiation and, in this case, it corresponded to the peak directivity. As (9) requires a relevant computational cost, especially attending optimization processes, simplifications based on half-wavelength spacing as derived in [26] (p. 206) become necessary. Otherwise, as the particular scope of this work regarding planar arrays is to analyze the extension of optimized linear arrays, the use of (9) was adopted. In the case of developing an optimization strategy involving explicitly the directivity of planar arrays, the particularized case here described is mandatory.

## 3. Results

In the following, all the described examples are based on linear and planar arrays with interelement spacings of λ/2, and regarding the optimization stage, coefficients of the cost function implemented to obtain the optimized values in each case were $c_{1}=4,\;c_{2}=2000,\;\mathrm{and}\;c_{3}=100$. The values of these coefficients have been set after tuning of the parameters to obtain the results reported in this work.

### 3.1. Fixing One Null

In order to study the variation in the SLL, an optimized array achieving the lowest SLL and maximizing directivity, without fixing any nulling direction, was defined as the reference. After this, a sweep was made, fixing one null every $2^{\circ },$ from $2^{\circ }$ to $88^{\circ },$ keeping the directivity to the one of the reference array. This process has been conducted for two different sized arrays: one with 40 elements and another with 80 elements.

#### 3.1.1. 40-Element Linear Arrays

The optimized reference array, calculated without fixing any nulls, resulted in a SLL of −17.27 dB, with a normalized peak directivity of 0.9 (which corresponds to 15.56 dB and 36 elements turned on).

**Figure 4:**
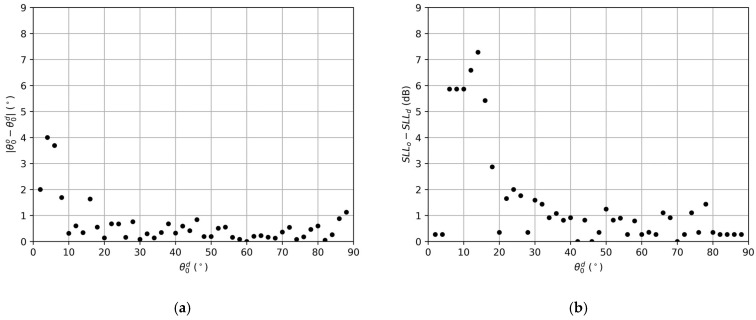
Results for the null fixing sweep for the linear array of 40 elements: (**a**) Deviation of the closest null in the pattern to the desired nulling direction; (**b**) Difference between the obtained SLL in every linear array of the sweep and the SLL of the reference linear array.

In the sweep, all the obtained arrays were able to fix the same desired directivity as the reference array. The distance between the desired nulling direction and the achieved one, for every calculated direction, is shown in Figure 4a. The SLL variation with the one achieved in the reference array is reported in Figure 4b. 

The average distance between the desired null and the obtained one was $0.63^{\circ },$ with a standard deviation of $0.83^{\circ }.$ The average difference between the obtained SLL and the reference one was 1.49 dB, with a standard deviation of 1.98 dB. The high deviations obtained for cases near the edge of the array pattern ($\theta <18^{\circ }$) report serious difficulties in fixing the angular positions of the nulls and lowering the SLL due to the last element of the array always being on, as explained in Section 2.4. Thus, it can be noted how fixing nulls near the edge of the radiation pattern, guaranteeing the same directivity and number of elements, which leads to a null filling of worst performance. On the other hand, fixing nulls near the central region of the pattern is also difficult due to the vicinity to the main beam. Therefore, we restricted this analysis to a more realistic range from $18^{\circ }$ to $84^{\circ }$ as the average distance between the desired null and the obtained one was $0.34^{\circ },$ with a standard deviation of $0.23^{\circ }$, and the average difference between the obtained SLL and the reference one was 0.82 dB, with a standard deviation of 0.65 dB.

#### 3.1.2. 80-Element Linear Array

The optimized reference array, calculated without fixing any nulls, presents a SLL of −19.85 dB, and a normalized peak directivity of 0.83 (which corresponds to 18.20 dB and 66 elements turned on).

**Figure 5:**
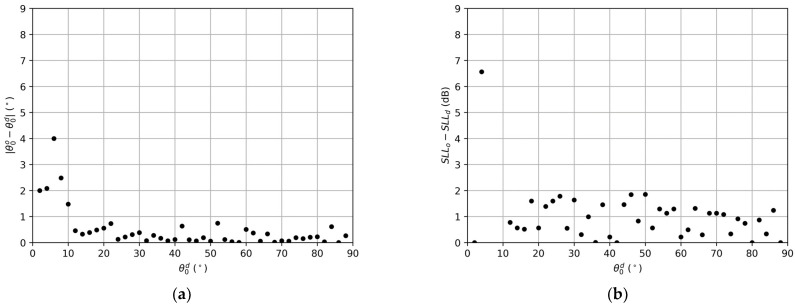
Results for the null fixing sweep for the linear array of 80 elements. (**a**) Deviation of the closest null in the pattern to the desired nulling direction. (**b**) Difference between the obtained SLL in every linear array of the sweep and the SLL of the reference linear array.

In the sweep, all the obtained arrays were able to fix the same desired directivity as the reference array. The distance between the desired nulling direction and the achieved one, for every calculated direction is shown in Figure 5a. The SLL variation with the one achieved in the reference array is reported in Figure 5b. 

The average distance between the desired null and the obtained one was $0.5^{\circ },$ with a standard deviation of $0.78^{\circ }.$ The average difference between the obtained SLL and the reference one was 1.77 dB, with a standard deviation of 3.07 dB. As for the previous example, if we restrict the calculation to the best working region of the sweep (which in this case was from $12^{\circ }$ to $88^{\circ }$), now the average distance between the desired null and the obtained one is $0.25^{\circ },$ with a standard deviation of $0.21^{\circ },$ and the average difference between the obtained SLL and the reference one is 0.88 dB, with a standard deviation of 0.56 dB.

### 3.2. Fixing Multiple Nulls

**Figure 6:**
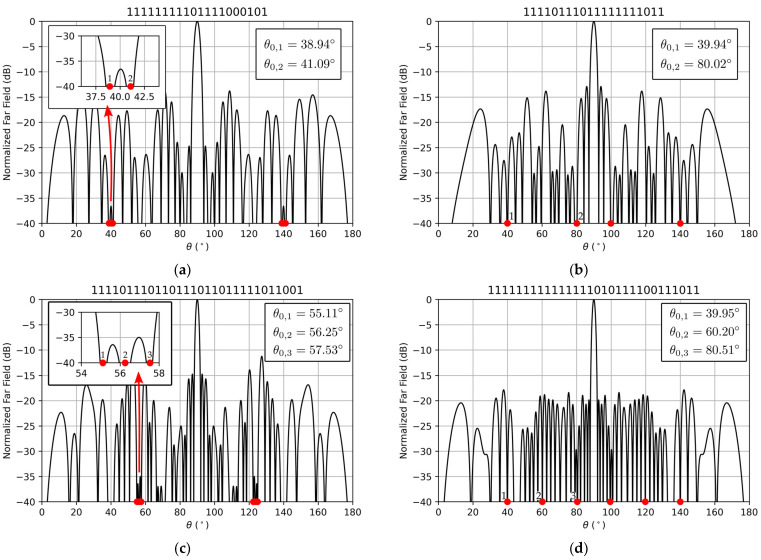
Examples of patterns with the achieved multiple nulling directions represented with red dots: (**a**) Two deep nulls close together at $38.94^{\circ }$ and $41.09^{\circ }$; (**b**) Two deep nulls separated at $39.94^{\circ }$ and $80.02^{\circ }$; (**c**) Three deep nulls close together at $55.11^{\circ }$, $56.25^{\circ }$, and $57.53^{\circ }$; (**d**) Three deep nulls separated at $39.95^{\circ }$, $60.20^{\circ }$, and $80.51^{\circ }$. The patterns with two deep nulls were generated by a linear array of 40 elements, while the patterns with three deep nulls were generated by a linear array of 60 elements. The right side of the symmetrical relative excitation vector of the elements is shown at the top of each generated pattern. Only the values for the deep nulls at the left side of the pattern are explicitly shown due to the symmetry of the pattern. The angular positions for the symmetrical nulls are obtained by $\theta_{0,i}^{sym}=180^{\circ }-\theta_{0,i}$.

By introducing multiple terms in the sum of the nulling directions cost, we can fix more than one deep null in our pattern. Four examples are shown in Figure 6, fixing two and three nulling directions, close together, and with a wide separation between each desired deep null.

Examples shown in Figure 6a,b consist of 40-element arrays. In the first example, nulls at positions $39^{\circ }$ and $41^{\circ }$ were desired, and the closest achieved nulling directions were $38.94^{\circ }$ and $41.09^{\circ }$, respectively. The normalized peak directivity of the resulting pattern was 0.75 (which corresponds to 14.77 dB and 30 elements turned on) and the SLL was −13.78 dB. In the example from Figure 6b, the desired nulling directions were $40^{\circ }$ and $80^{\circ }$, and deep nulls at $39.94^{\circ }$ and $80.02^{\circ }$ were achieved, with a normalized peak directivity of 0.85 (which corresponds to 15.31 dB and 30 elements turned on), resulting in a SLL of −12.9 dB.

**Figure 7:**
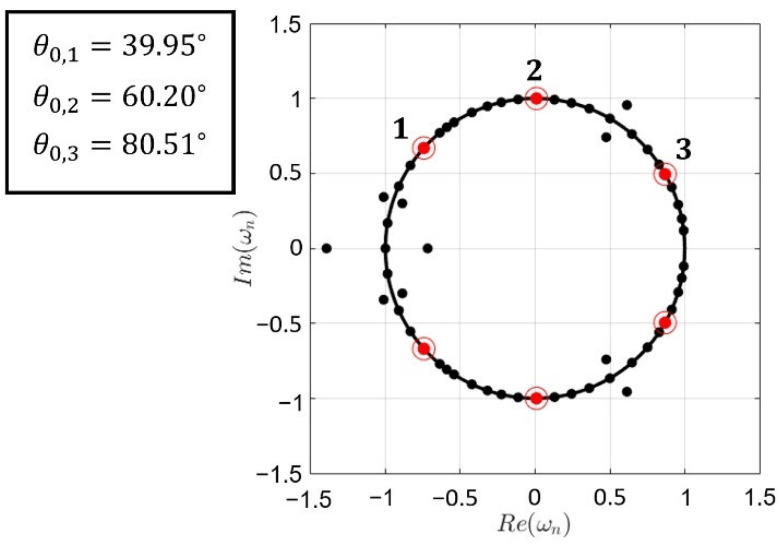
Representation by means of the Schelkunoff unit circle of the roots of the relative excitation distribution associated with the pattern of Figure 6d. The angular positions of the deep nulls 1–3, which generates the far-field radiation pattern of Figure 6d in this representation corresponds to $\psi_{0,n}=\pi \cdot cos\left(\theta_{0,n}\right).$ Only the values for the deep nulls at the upper side of the unit circle are explicitly reported due to the symmetry of the pattern. The angular positions for the symmetrical nulls are obtained by $\theta_{0,i}^{sym}=180^{\circ }-\theta_{0,i}$.

Figure 6c,d correspond to 60-element arrays with three desired nulling directions. In the example shown in Figure 6c, deep nulls at angles of $55^{\circ }$, $56^{\circ }$, and $57^{\circ }$ were desired, and the closest obtained nulling directions were $55.11^{\circ }$, $56.25^{\circ }$ and $57.53^{\circ }$, with a normalized peak directivity of 0.73 (which corresponds to 16.41 dB and 44 elements turned on) and a SLL of −11.24 dB. In the pattern from Figure 6d, three separated deep nulls at $40^{\circ }$, $60^{\circ }$, and $80^{\circ }$ were desired, and nulls at angles of $39.95^{\circ }$, $60.20^{\circ }$, and $80.51^{\circ }$ were achieved. A graphical representation of the roots of the pattern with the Schelkunoff unit circle, highlighting the ones corresponding to the fixed deep nulls lying on the unit circle, is reported in Figure 7. The normalized peak directivity in this case was 0.83 (which corresponds to 16.97 dB and 50 elements turned on) and the SLL = −17.84 dB.

The roots of the radiation pattern from Figure 6d, shown in Figure 7, are either exactly on the Schelkunoff unit circle, or are given by pairs, with the same $\psi_{0,n}$, one inside and one outside. The study made in [30] addressed this, concluding on the existence of a multiplicity of solutions, corresponding to different relative excitation vectors of the array, resulting in the same contributions to the amplitude of the radiation pattern. Although this is applicable in our case, it has been checked that the case here reported is the only one resulting in real, symmetric values, consisting of ones and zeros for the relative excitation vector.

### 3.3. Binary GA Comparison

To develop a crosscheck and comparing the performance of the present method, a binary GA algorithm [11,31] has been introduced in the same optimization strategy including the same null fixing method and cost function (6). Both optimizations were encoded in MATLAB R2021b by Mathworks Inc. (Natick, Apple Hill Campus, MA, USA) and all calculations were performed in a personal computer with an AMD A10-9600P processor running at 2.40 GHz and 12 GB of RAM.

The obtained antenna arrays with the GA presented similar deviations from the desired characteristics of the pattern in all the mentioned examples, with a population size of 350, single-point crossover with a crossover fraction of 0.8, roulette selection, mutation of bits with a probability of 0.01 and one elite individual per generation.

Although this method a priori represents the most natural way of facing thinned linear arrays of uniform relative excitations, due to its binary nature, it results in a considerable time difference with the hybrid SA. Similar running times can sometimes be achieved for small arrays up to 40 elements in size, as shown in Table 1, but our experimentation showed a great increase in the difference for arrays with more elements, up to a factor of approximately nine times the running time of the SA for a 200-element array.

### 3.4. Planar Arrays with Separable Distribution

Extending the result obtained for two 40-element linear arrays, each one optimized by the method here reported fixing two different nulling directions, so we calculated the relative excitation pattern for a planar array with a rectangular grid and boundary with a separable distribution. 

For the x-axis ($\varphi =0^{\circ }$), an array with desired nulling directions in $\theta_{0,1}^{d}=40^{\circ }$ and $\theta_{0,2}^{d}=\;45^{\circ }$ was calculated, achieving deep nulls at $\theta_{0,1}^{o}=39.61^{\circ }$ and $\theta_{0,2}^{o}=\;45.02^{\circ }$. The normalized peak directivity of this pattern was 0.85 (which corresponds to 15.31 dB and 34 turned on) and the SLL was −16.02 dB. For the y-axis ($\varphi =90^{\circ }$), an array with desired nulling directions in $\theta_{0,1}^{d}=50^{\circ }$ and $\theta_{0,2}^{d}=\;60^{\circ }$ was synthesized, obtaining deep nulls at $\theta_{0,1}^{o}=\;50.03^{\circ }$ and $\theta_{0,2}^{o}=\;60.00^{\circ }$ with a normalized peak directivity of 0.9 (which corresponds to 15.56 dB and 36 turned on) and a SLL of −16.37 dB. Both relative excitation vectors for the arrays are shown in Table 2.

**Figure 8:**
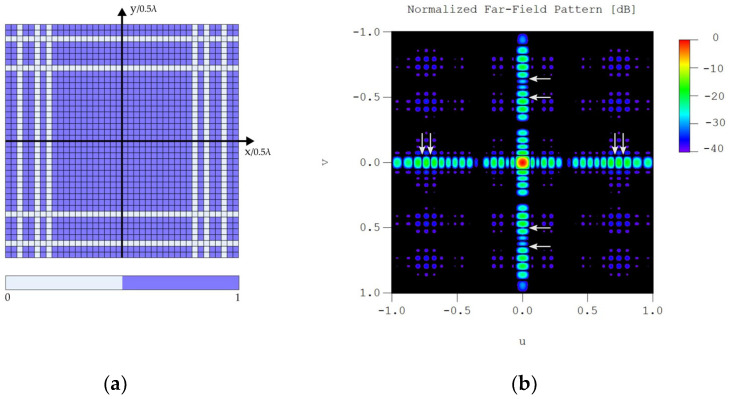
(**a**) Relative excitation pattern for the elements of the planar array with rectangular grid and boundary with separable distribution. (**b**) Normalized far-field radiation pattern of the planar array from Figure 8a with the achieved deep nulls represented with arrows.

The corresponding relative excitation pattern for the planar array generated by the separable distribution procedure is shown in Figure 8a, while its corresponding normalized far-field radiation pattern is shown in Figure 8b. 

We must highlight the fact that our linear arrays are optimized lying on the z-axis, so when we produce the planar array on the x−y plane, the null positions of our linear patterns are shifted $90^{\circ }$ in the θ coordinate, aside from one of them (the one chosen to be in the y-axis) turned $90^{\circ }$ around the z-axis, in the φ coordinate. The radiation diagram shown in Figure 8b uses the coordinate system u=sin(θ)cos(φ) and v=sin(θ)sin(φ), so with the described shifts resulting from the movement of the linear arrays, our deep nulls are now at $\left(u_{0,1}^{x},v_{0,1}^{x}\right)=\left(\pm 0.7069,0\right)$, $\left(u_{0,2}^{x},v_{0,2}^{x}\right)=\left(\pm 0.7705,0\right)$, $\left(u_{0,1}^{y},v_{0,1}^{y}\right)=\left(0,\pm 0.5000\right),$ and $\left(u_{0,2}^{y},v_{0,2}^{y}\right)=\left(0,\pm 0.6424\right)$, where the plus and minus sign comes from the symmetry of the linear array patterns. The resulting far field pattern from the planar array presents a SLL of −16.37 dB, and a normalized peak directivity of 0.69 (which corresponds to a peak directivity of 35.35 dB and 1224 elements turned on), where the peak directivity of the uniformly excited planar array of the same size was used in the normalization.

## 4. Discussion

In the present work, an innovative method for fixing deep nulls in radiation patterns of symmetrical linear arrays based on the hybrid SA global optimization algorithm was implemented. This method is able to synthesize required radiation patterns restricting the possible relative excitation values for the arrays to a binary possibility of zero or one, facilitating the feeding network implementation. To the best knowledge of the authors, this approach represents the first achievement of simultaneously including both array thinning and deep null fixing in the array pattern synthesis framework.

The algorithm shows a great ability for fixing a single nulling direction in a big range of the pattern, maintaining a fixed desired directivity, while keeping the SLL close to the one from the optimized array without any fixed deep nulls. In the case of the 40-element array, an average deviation of $0.34^{\circ }$ from the desired nulling direction was achieved in the range $\theta \in \left[18^{\circ },84^{\circ }\right]$, and the average difference between the obtained SLL and the reference one was 0.82 dB. For the 80-element array, an average deviation of $0.25^{\circ }$ from the desired nulling direction was achieved in the range $\theta \in \left[12^{\circ },88^{\circ }\right]$, and the average difference between the obtained SLL and the reference one in this case was 0.88 dB.

In order to show the flexibility of the procedure, examples with multiple nulling directions have been also analyzed, both in angles close together and presenting a large separation between them. In such a case, it is important to highlight the increased difficulty for each added deep null, resulting in worst fixing precision or higher SLL of the pattern produced by the method.

Regarding computational costs, it can be concluded that running time differences between the present methodology and GA-based alternatives increase considerably with the number of elements of the arrays.

An extension for fixing certain nulling directions in planar arrays, while keeping the relative excitations of the antenna array elements binary (zeros and ones) was implemented by using separable distributions, obtaining the desired radiation patterns with fixed nulls in the $\varphi =0^{\circ }$ and $\varphi =90^{\circ }$ planes. This method leads to the desired nulling patterns in the mentioned planes, but reduced SLL elsewhere with a consequent beam broadening and reduced directivity. Separable distributions are also strictly only applicable to planar arrays with rectangular grids and boundaries, limiting their use.

As future developments for the strategy regarding linear array approaches, studies involving non-symmetrical solutions are under consideration. To this aim, it is important to highlight that for a sum pattern, as its amplitude distribution is always symmetrical, an asymmetrical phase distribution becomes mandatory in addressing an asymmetrical nature of the pattern [26] (p. 167). Therefore, the envisaged idea is based on the introduction of an asymmetrical phase distribution within the present methodology to improve its flexibility.

Additionally, an alternative planar extension also under consideration is based on the collapsed distributions paradigm [29,32]. More precisely, the implementation of relative excitations for the planar array with 0 and ±1 as boundary conditions can be proposed as a working hypothesis. In such a way, a two-bit basis for the feeding network could be managed: one bit regarding relative amplitudes (0 and 1), and another bit for relative phases ($0^{\circ }$ and $180^{\circ }$) of the active elements. A second optimization stage is here proposed, after the generation of the linear arrays, looking for the individual relative excitation values for each element of the planar array in order to achieve, as collapsed distributions at certain angles, the linear arrays from the first optimization. In such a way, the invocation of the principle of collapsed distributions will guarantee the implementation of a methodology to project relative excitations of 1 or 0 for the equivalent linear arrays as required.

## Figures and Tables

**Table 1:** Comparison of the average execution times for each implemented algorithm.

Algorithm	Average Execution Time (s)			
	1 Deep Null		2 Deep Nulls	3 Deep Nulls
	40-Element Array	80-Element Array	40-Element Array	60-Element Array
SA	45.84	314.41	111.39	130.03
GA	111.48	562.79	134.98	255.36

**Table 2:** Right side of the symmetrical relative excitation vector for the linear arrays used in the design of the planar separable distribution.

$\mathrm{Array}\;\mathrm{in}\;\varphi =0^{\circ }$	$\mathrm{Array}\;\mathrm{in}\;\varphi =90^{\circ }$
11111111111101011011	11111111111101111011

## Data Availability

Data are contained within the article.
